# Supplementary figures and images for: Prevalence and factors associated with chronic use of levothyroxine: A cohort study
Source: PLoS One. 2021 Dec 20;16(12):e0261160. doi: 10.1371/journal.pone.0261160 (PMC8687586; doi:10.1371/journal.pone.0261160)

**S1 Fig: Flow chart**

**
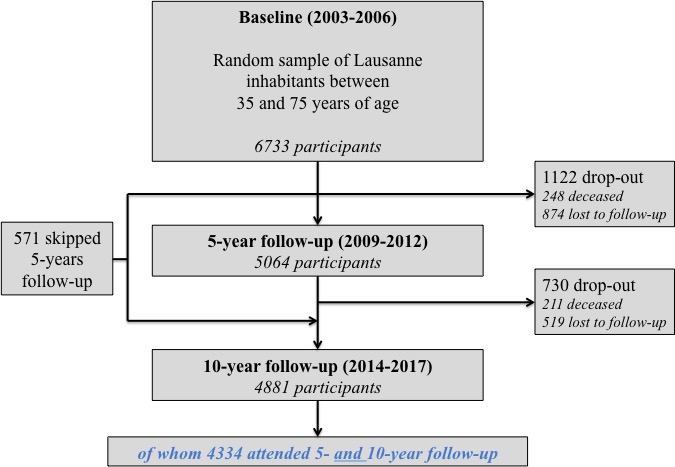
**

Supplement: S1 Fig — (DOCX) [file pone.0261160.s001.docx]
