# Supplementary material for: Prevalence and factors associated with chronic use of levothyroxine: A cohort study
Source: PLoS One. 2021 Dec 20;16(12):e0261160. doi: 10.1371/journal.pone.0261160 (PMC8687586; doi:10.1371/journal.pone.0261160)
Supplement: S1 Table — aother than levothyroxine. Abbreviations: SD: standard deviation; n: number of participants; p25-p75: 25th– 75 percentile; BMI: Body Mass Index; TSH: Thyroid Stimulating Hormone. (DOCX) [file pone.0261160.s002.docx]

**S1 Table: Demographic characteristics at baseline comparing participants who attended 5- and 10-year follow up (n=4334) and participants lost to follow up (n=2399)**

|  | **Lost to follow-up or deceased (n=2399)** | **Attended 5- and 10-year follow-up (n=4334)** | **p for difference** | |
| --- | --- | --- | --- | --- |
| **Age (years)** |  |  |  | |
| Mean (SD) | **53.9 (11.2)** | **51.9 (10.4)** | **< 0.001** | |
| Range | **34.9 – 75.4** | **34.9 - 75.4** |  | |
| **Female sex - % (n)** | **48.4 (1162)** | **54.9 (2382)** | **< 0.001** | |
| **BMI (kg/m^2^) - mean (SD)** | **26.3 (4.8)** | **25.5 (4.3)** | **< 0.001** | |
| **N. of drugs^a^- median (p25-p75)** | **2.1 (0-3)** | **2.0 (0-4)** | **0.85** | |
| **Hypertension - % (n)** | **42.6 (1019)** | **31.5 (1366)** | **< 0.001** |  |
| **Diabetes - % (n)** | **9.2 (219)** | **5.0 (217)** | **< 0.001** | |
| **Current smoking - % (n)** | **31.2 (749)** | **24.5 (1063)** | **< 0.001** | |
| **Lipid lowering drug - % (n)** | **13.6 (325)** | **10.6 (460)** | **< 0.001** | |
| **Family history of  thyroid pathologies - % (n)** | **5.5 (132)** | **7.9 (343)** | **< 0.001** | |
| **TSH (mIU/l) - mean (SD)** | **2.6 (3.6)** | **2.5 (2.8)** | **0.616** | |

*^a^other than levothyroxine*

*Abbreviations:* ***SD****: standard deviation;* ***n****: number of participants;* ***p25-p75****: 25^th^ – 75 percentile****; BMI:*** *Body Mass Index;* ***TSH****: Thyroid Stimulating Hormone*
