# Supplementary material for: Prevalence and factors associated with chronic use of levothyroxine: A cohort study
Source: PLoS One. 2021 Dec 20;16(12):e0261160. doi: 10.1371/journal.pone.0261160 (PMC8687586; doi:10.1371/journal.pone.0261160)
Supplement: S2 Table — acombination drug. (DOCX) [file pone.0261160.s003.docx]

**S2 Table: Sensitivity Analysis including participants who only answered drug questionnaire at one follow-up – Ranking of the most used chronic drugs from the CoLaus cohort**

|  | **DRUG** |  |
| --- | --- | --- |
| 1 | Aspirin | |
| 2 | **Levothyroxine** | |
| 3 | Simvastatin | |
| 4 | Calcium+Vit D^a^ | |
| 5 | Atorvastatin | |
| 6 | Metformin | |
| 7 | Metoprolol | |
| 8 | Candesartan | |
| 9 | Zolpidem | |
| 10 | Pravastatin | |
| 11 | Lisinopril | |
| 12 | Paracetamol | |
| 13 | Amlodipin | |
| 14 | Esomeprazole | |
| 15 | Acenocoumarol | |
| 16 | Omeprazole | |
| 17 | Estradiol | |
| 18 | Allopurinol | |
| 19 | Bisoprolol | |
| 20 | Lorazepam | |
| 21 | Irbesartan and hydrochlorothiazide^a^ | |
| 22 | Atenolol | |
| 23 | Chondroitin sulfate | |
| 24 | Torasemide | |
| 25 | Citalopram | |
| 26 | Perindopril | |
| 27 | Rosuvastatin | |
| 28 | Pantoprazole | |
| 29 | Losartan | |
| 30 | Enalapril | |

*^a^combination drug*
