# Supplementary material for: Prevalence and factors associated with chronic use of levothyroxine: A cohort study
Source: PLoS One. 2021 Dec 20;16(12):e0261160. doi: 10.1371/journal.pone.0261160 (PMC8687586; doi:10.1371/journal.pone.0261160)
Supplement: S3 Table — a Paracetamol would rank first according to the invoice data. However, in the analysis of the CoLaus data we chose to omit on-demand and over-the-counter drugs. This exclusion could not be done with the SantéSuisse data, we therefore chose not to report paracetamol in the first position. b combination drug; HCT = hydrochlorothiazide Ranking of most invoiced chronic drugs using insurance data of 2014–2018 from SASIS, analysed by SantéSuisse, based on number of pills invoiced per insured person per year in Switzerland. (DOCX) [file pone.0261160.s004.docx]

**S3 Table: Ranking of the most used drugs in Switzerland**

|  | **2018** | **2017** | **2016** | **2015** | **2014** |
| --- | --- | --- | --- | --- | --- |
| 1^a^ | Aspirin | Aspirin | Aspirin | Aspirin | Aspirin |
| 2 | Calcium+Vit D1^b^ | Calcium+Vit D1^b^ | Calcium+Vit D1^b^ | Calcium+Vit D1^b^ | Calcium+Vit D1^b^ |
| 3 | **Levothyroxine** | **Levothyroxine** | Atorvastin | **Levothyroxine** | Metformin |
| 4 | Atorvastatin | Atorvastin | **Levothyroxine** | Metformin | **Levothyroxine** |
| 5 | Metformin | Metformin | Metformin | Atorvastin | Atorvastin |
| 6 | Metoprolol | Metoprolol | Metoprolol | Torasemide | Torasemide |
| 7 | Torasemide | Torasemide | Torasemide | Metoprolol | Metoprolol |
| 8 | Amlodipin | Amlodipin | Amlodipin | Amlodipin | Amlodipin |
| 9 | Bisoprolol | Bisoprolol | Bisoprolol | Bisoprolol | Lorazepam |
| 10 | Rosuvastatin | Chondroitin sulfate | Esomeprazole | Lorazepam | Bisoprolol |
| 11 | Chondroitin sulfate | Esomeprazole | Chondroitin sulfate | Esomeprazole | Esomeprazole |
| 12 | Esomeprazol | Rosuvastatin | Lorazepam | Chondroitin sulfate | Chondroitin sulfate |
| 13 | Lisinopril | Lorazepam | Rosuvastatin | Lisinopril | Lisinopril |
| 14 | Lorazepam | Lisinopril | Lisinopril | Rosuvastatin | Zolpidem |
| 15 | Candesartan | Candesartan | Zolpidem | Zolpidem | Rosuvastatin |
| 16 | Zolpidem | Zolpidem | Candesartan | Candesartan | Simvastatin |
| 17 | Allopurinol | Allopurinol | Simvastatin | Citalopram | Candesartan |
| 18 | Omeprazole | Simvastatin | Allopurinol | Simvastatin | Omeprazole |
| 19 | Simvastatin | Omeprazole | Omeprazole | Omeprazole | Citalopram |
| 20 | Perindopril | Prevastatin | Prevastatin | Allopurinol | Allopurinol |
| 21 | Citalopram | Citalopram | Citalopram | Prevastatin | Prevastatin |
| 22 | Pravastatin | Perindopril | Acenocoumarol | Acenocoumarol | Acenocoumarol |
| 23 | Irbesartan and HCT^b^ | Acenocoumarol | Perindopril | Perindopril | Atenolol |
| 24 | Acenocoumarol | Irbesartan and HCT^b^ | Irbesartan and HCT^b^ | Atenolol | Perindopril |
| 25 | Estradiol | Atenolol | Atenolol | Irbesartan and HCT^b^ | Irbesartan and HCT^b^ |
| 26 | Fluoxetine | Estradiol | Estradiol | Enalapril | Fluoxetine |
| 27 | Atenolol | Fluoxetine | Fluoxetine | Losartan | Enalapril |
| 28 | Losartan | Losartan | Losartan | Fluoxetine | Losartan |
| 29 | Enalapril | Enalapril | Enalapril | Estradiol | Estradiol |
| 30 | Calcium+Vit D2^b^ | Calcium+Vit D2^b^ | Calcium+Vit D2^b^ | Calcium+Vit D2^b^ | Calcium+Vit D2^b^ |

*^a^ Paracetamol would rank first according to the invoice data. However, in the analysis of the CoLaus data we chose to omit on-demand and over-the-counter drugs. This exclusion could not be done with the SantéSuisse data, we therefore chose not to report paracetamol in the first position.*

*^b^ combination drug;* ***HCT****=hydrochlorothiazide*

*Ranking of most invoiced chronic drugs using insurance data of 2014-2018 from SASIS, analysed by SantéSuisse, based on number of pills invoiced per insured person per year in Switzerland.*
