# Supplementary material for: Prevalence and factors associated with chronic use of levothyroxine: A cohort study
Source: PLoS One. 2021 Dec 20;16(12):e0261160. doi: 10.1371/journal.pone.0261160 (PMC8687586; doi:10.1371/journal.pone.0261160)
Supplement: S4 Table — aother than levothyroxine. Results are expressed as odds ratio and (95% confidence interval) To assess the association with the categorical variables age, BMI and number of drugs respectively, we included the categorial variable in the multivariable model instead of the continuous related one (e.g. the model with BMI as categorical has the following variables: BMI categories, age (continuous), sex, N. drugs (continuous), hypertension, diabetes, current smoking, lipid lowering drugs, family history, TSH, handgrip). Abbreviations: iqr: interquartile range, SD: standard deviation, n: number of participants; p25-p75: 25th– 75th percentile, BMI: Body Mass Index. TSH: Thyroid Stimulating Hormone. Definition of BMI categories: underweight (<18.5), normal (18.5–24.9), overweight (25–29.9), obese (≥30). (DOCX) [file pone.0261160.s005.docx]

**S4 Table: Association between chronic levothyroxine use and each factor at a time from the multivariable models including categorical variables**

|  | **Model with age as categorical variable** | **p-value** | **Model with BMI as categorical variable** | **p-value** | **Model with N. drugs as categorical variable** | **p-value** |
| --- | --- | --- | --- | --- | --- | --- |
| **Age (per 1-year increase)** |  |  | **1.03 (1.01-1.05)** | **<0.01** | **1.03 (1.01-1.06)** | **<0.01** |
| **Age categories** |  |  |  |  |  |  |
| **< 55 years** | reference |  |  |  |  |  |
| **≥55 and <65 years** | 1.19 (0.68-2.10) | 0.53 |  |  |  |  |
| **≥65 and <75 years** | **2.00 (1.16-3.45)** | **0.01** |  |  |  |  |
| **≥75 years** | **2.47 (1.31-4.67)** | **<0.01** |  |  |  |  |
| **BMI (per 1-unit increase)** | **1.05 (1.02-1.09)** | **<0.01** |  |  | **1.06 (1.03-1.10)** | **<0.01** |
| **BMI categories** |  |  |  |  |  |  |
| **underweight** |  |  | 0.35 (0.47-2.69) | 0.32 |  |  |
| **normal** |  |  | reference |  |  |  |
| **overweight** |  |  | 1.45 (0.96-2.21) | 0.07 |  |  |
| **obese** |  |  | **2.14 (1.34-3.42)** | **<0.01** |  |  |
| **N. drugs*^a^* (per 1-unit increase)** | **1.22 (1.16-1.29)** | **<0.01** | **1.22 (1.16-1.29)** | **<0.01** |  |  |
| **N. drugs categories** |  |  |  |  |  |  |
| **<5** |  |  |  |  | reference |  |
| **5-9** |  |  |  |  | 1.27 (0.83-1.94) | 0.27 |
| **≥10** |  |  |  |  | **3.35 (1.77-6.34)** | **<0.01** |
| **Female sex vs male** | **11.96 (5.28-27.05)** | **<0.01** | **12.18 (5.37-27.61)** | **<0.01** | **11.48 (5.07-26.00)** | **<0.01** |
| **Hypertension (yes vs no)** | 0.90 (0.61-1.34) | 0.61 | 0.90 (0.60-1.34) | 0.60 | 1.09 (0.74-1.63) | 0.65 |
| **Diabetes (yes vs no)** | 0.66 (0.35-1.22) | 0.18 | 0.67 (0.36-1.23) | 0.20 | 0.82 (0.45-1.50) | 0.52 |
| **Current smoking (yes vs no)** | 0.72 (0.42-1.23) | 0.23 | 0.71 (0.41-1.22) | 0.21 | 0.76 (0.44-1.29) | 0.31 |
| **Lipid lowering drug (yes vs no)** | 0.79 (0.52-1.20) | 0.27 | 0.80 (0.53-1.21) | 0.29 | .03 (0.68-1.56 ) | 0.89 |
| **Family history of thyroid pathologies (yes vs no)** | **2.19 (1.37-3.50)** | **<0.01** | **2.19 (1.37-3.49)** | **<0.01** | **2.21 (1.40-3.50)** | **<0.01** |
| **TSH (per 1-unit increase)** | 1.01 (0.95-1.08) | 0.66 | 1.01 (0.95-1.08) | 0.77 | 1.01 (0.94-1.73) | 0.86 |
| **Handgrip (per 1-kg increase)** | 1.01 (0.98-1.04) | 0.68 | 1.01 (0.98-1.04) | 0.73 | 1.00 (0.97-1.03) | 0.81 |

*^a^other than levothyroxine.*

*Results are expressed as odds ratio and (95% confidence interval)*

*To assess the association with the categorical variables age, BMI and number of drugs respectively, we included the categorial variable in the multivariable model instead of the continuous related one (e.g. the model with BMI as categorical has the following variables: BMI categories, age (continuous), sex, N. drugs (continuous), hypertension, diabetes, current smoking, lipid lowering drugs, family history, TSH, handgrip). Abbreviations:* ***iqr****: interquartile range,* ***SD****: standard deviation,* ***n****: number of participants;* ***p25-p75****: 25^th^ – 75t^h^ percentile,* ***BMI****: Body Mass Index.* ***TSH****: Thyroid Stimulating Hormone. Definition of BMI categories: underweight (<18.5), normal (18.5-24.9), overweight (25-29.9), obese (≥30)*
